# Decadal trends in ^137^Cs concentrations in the bark and wood of trees contaminated by the Fukushima nuclear accident

**DOI:** 10.1038/s41598-022-14576-1

**Published:** 2022-07-04

**Authors:** Shinta Ohashi, Katsushi Kuroda, Hisashi Abe, Akira Kagawa, Masabumi Komatsu, Masaki Sugiyama, Youki Suzuki, Takeshi Fujiwara, Tsutomu Takano

**Affiliations:** 1 Department of Wood Properties and Processing, Forestry and Forest Products Research Institute (FFPRI), 1 Matsunosato, Tsukuba, Ibaraki 305-8687 Japan; 2 Department of Mushroom Science and Forest Microbiology, FFPRI, 1 Matsunosato, Tsukuba, Ibaraki 305-8687 Japan; 3 Hokkaido Research Center, FFPRI, 7 Hitsujigaoka, Toyohira, Sapporo , Hokkaido 062-8516 Japan; 4 Center for Forest Restoration and Radioecology, FFPRI, 1 Matsunosato, Tsukuba, Ibaraki 305-8687 Japan

**Keywords:** Environmental sciences, Forest ecology, Forestry

## Abstract

Understanding the actual situation of radiocesium (^137^Cs) contamination of trees caused by the Fukushima nuclear accident is essential for predicting the future contamination of wood. Particularly important is determining whether the ^137^Cs dynamics within forests and trees have reached apparent steady state. We conducted a monitoring survey of four major tree species (Japanese cedar, Japanese cypress, konara oak, and Japanese red pine) at multiple sites. Using a dynamic linear model, we analyzed the temporal trends in ^137^Cs activity concentrations in the bark (whole), outer bark, inner bark, wood (whole), sapwood, and heartwood during the 2011–2020 period. The activity concentrations were decay-corrected to September 1, 2020, to exclude the decrease due to the radioactive decay. The ^137^Cs concentrations in the whole and outer bark samples showed an exponential decrease in most plots but a flat trend in one plot, where ^137^Cs root uptake is considered to be high. The ^137^Cs concentration ratio (CR) of inner bark/sapwood showed a flat trend but the CR of heartwood/sapwood increased in many plots, indicating that the ^137^Cs dynamics reached apparent steady state within one year in the biologically active parts (inner bark and sapwood) and after several to more than 10 years in the inactive part (heartwood). The ^137^Cs concentration in the whole wood showed an increasing trend in six plots. In four of these plots, the increasing trend shifted to a flat or decreasing trend. Overall, the results show that the ^137^Cs dynamics within forests and trees have reached apparent steady state in many plots, although the amount of ^137^Cs root uptake in some plots is possibly still increasing 10 years after the accident. Clarifying the mechanisms and key factors determining the amount of ^137^Cs root uptake will be crucial for predicting wood contamination.

## Introduction

After the Fukushima Dai-ichi Nuclear Power Plant (FDNPP) accident in March of 2011, a wide area of forests in eastern Japan was contaminated with radionuclides. In particular, radiocesium (^137^Cs) has the potential to threaten the forestry and wood production in the contaminated area for many decades because it was released in large amounts (10 PBq)^1^ and has a relatively long half-life (30 years). Radiocesium levels for some wood uses are strictly regulated in Japan (e.g., 40 Bq kg^−1^ for firewood^2^ and 50 Bq kg^−1^ for mushroom bed logs^3^), meaning that multipurpose uses of wood from even moderately contaminated areas are restricted. Although a guidance level of radiocesium in construction wood has not been declared in Japan, the permissible levels in some European countries (370–740 Bq kg^−1^)^4–6^ suggest that logging should be precautionary within several tens of kilometers from the FDNPP, where the ^137^Cs activity concentration in wood potentially exceeds 1,000 Bq kg^−1^ [refs. ^7,8^]. To determine whether logging should proceed, the long-term variation in wood ^137^Cs concentration must be predicted as accurately as possible. Many simulation models successfully reproduce the temporal variations in the early phase after the FDNPP accident, but produce large uncertainties in long-term predictions^9^. To understand the ^137^Cs dynamics in forests and trees and hence refine the prediction models, it is essential to provide and analyze the observational data of ^137^Cs activity concentrations in tree stem parts.

Accident-derived ^137^Cs causes two types of tree contamination: direct contamination by ^137^Cs fallout shortly after the accident, and indirect contamination caused by surface uptake from directly contaminated foliage/bark^10,11^ and root uptake from contaminated soil^12^. The ^137^Cs concentration in bark that pre-exists the accident was affected by both ^137^Cs drop/wash off from bark surfaces and ^137^Cs uptake because the bark consists of a directly contaminated outer bark (rhytidome) and an indirectly contaminated inner bark (phloem). Given that the ^137^Cs content was 10 times higher in the outer bark than in the inner bark in 2012^13^ and the ^137^Cs concentration in the whole bark decreased during the 2011–2016 period at many study sites^8^, the temporal variation in the whole bark ^137^Cs concentration during the early post-accident phase must be mainly contributed by drop/wash off of ^137^Cs on the outer bark surface.

In contrast, stem wood (xylem) covered by bark was contaminated only indirectly. Although ^137^Cs distribution in sapwood (outer part of stem wood; containing living cells) and heartwood (inner part of stem wood; containing no living cells) is non-uniform and species-specific^8,13–15^, the ^137^Cs concentration in whole wood depends on the amount of ^137^Cs uptake. Because the dissolvable ^137^Cs on the foliar/bark surface decreased significantly within 2011^16^, the main route of ^137^Cs uptake since 2012 is likely root uptake rather than surface uptake. A monitoring survey during 2011–2016 showed that the temporal trend in the whole wood ^137^Cs concentration can be increasing, decreasing, or flat^8^, suggesting that ^137^Cs root uptake widely differs among sites and species.

Meanwhile, many simulation models have predicted an initial increase in the whole wood ^137^Cs concentration after the accident, followed by a gradual decline^9^. The initial increase is attributable to the increase in soil ^137^Cs inventory, and the following decline is mainly attributed to radioactive decay, dilution by wood biomass increment, and immobilization in the soil. Therefore, the trend shift from increasing to decreasing is a good indicator that shows the ^137^Cs dynamics within the forest have reached apparent steady state, which is characterized by slower changes in ^137^Cs concentration, bioavailability, and partitioning in the forest^12,17,18^. However, the timing of the trend shift predicted by the models have large uncertainty, varying from several years to a few decades from the accident^9^. Moreover, the trend shift has not been confirmed by observational data after the FDNPP accident. Although our monitoring survey cannot easily identify the key driving factors of the temporal trends, it can directly discern the trend shift from increasing to decreasing, and the timeframe of the increasing trend. The confirmation of the trend shift will accelerate the understanding of key factors of ^137^Cs root uptake, because important parameters such as transfer factor and CR are originally defined for a steady state condition^18^.

The present study aims to clarify the temporal trends of ^137^Cs concentrations in bark and wood of four major tree species (Japanese cedar, Japanese cypress, konara oak, and Japanese red pine) at multiple sites during the 10 years following the FDNPP accident. Detecting a trend shift from increasing to decreasing in the wood ^137^Cs concentration was especially important to infer whether the ^137^Cs dynamics within the forest have reached apparent steady state. We update Ohashi et al.^8^, who analyzed the monotonous increasing or decreasing trends during 2011–2016, with observational data of 2017–2020 and a more flexible time-series analysis using a dynamic linear model (DLM). The DLM is suitable for analyzing data including observational errors and autocorrelation, and has the advantage of being applicable to time-series data with missing years. For a more detailed understanding of bark contamination and the ^137^Cs dynamics in tree stems, we also newly provide data on the ^137^Cs concentrations in the outer and inner barks. The temporal trends in the ^137^Cs CRs of outer bark/inner bark, heartwood/sapwood, and inner bark/sapwood were analyzed to confirm whether the ^137^Cs dynamics within the trees have reached apparent steady state.

## Materials and methods

### Monitoring sites and species

**Figure 1:**
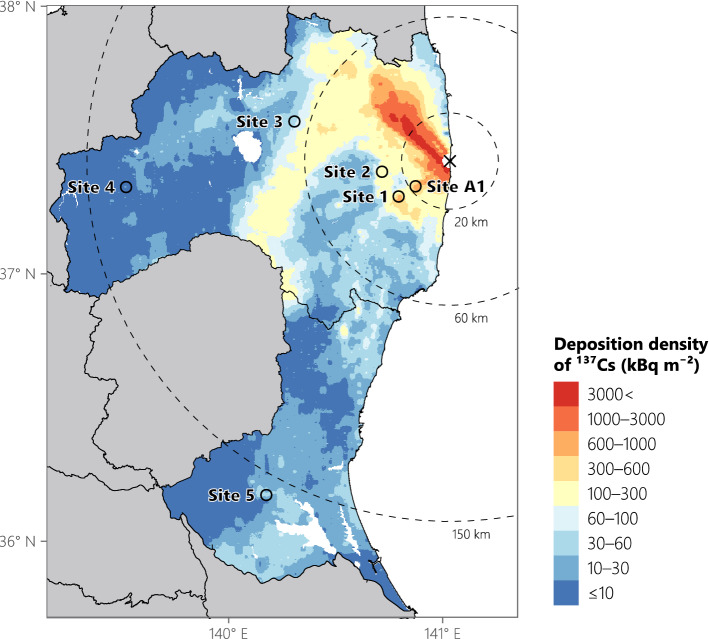
Locations of the monitoring sites and initial deposition densities of ^137^Cs (decay-corrected to July 2, 2011) following the Fukushima nuclear accident in Fukushima and Ibaraki Prefectures. *Open circles* indicate the monitoring sites and the *cross mark* indicates the Fukushima Dai-ichi Nuclear Power Plant. Data on the deposition density were provided by MEXT^19,20^ and refined by Kato et al.^21^. The map was created using R (version 4.1.0)^22^ with ggplot2 (version 3.3.5)^23^ and sf (version 1.0–0)^24^ packages.

The monitoring survey was conducted at five sites in Fukushima Prefecture (sites 1–4 and A1) and at one site in Ibaraki Prefecture (site 5), Japan (Fig. 1). Sites 1, 2, and A1 are located in Kawauchi Village, site 3 in Otama Village, site 4 in Tadami Town, and site 5 in Ishioka City. Monitoring at sites 1–5 was started in 2011 or 2012, and site A1 was additionally monitored since 2017. The tree species, age, mean diameter at breast height, initial deposition density of ^137^Cs, and sampling year of each sample at each site are listed in Table 1. The dominant tree species in the contaminated area, namely, Japanese cedar (*Cryptomeria japonica* [L.f.] D.Don), Japanese cypress (*Chamaecyparis obtusa* [Siebold et Zucc.] Endl.), konara oak (*Quercus serrata* Murray), and Japanese red pine (*Pinus densiflora* Siebold et Zucc.) were selected for monitoring. Japanese chestnut (*Castanea crenata* Siebold et Zucc.) was supplementally added in 2017. The cedar, cypress, and pine are evergreen coniferous species, and the oak and chestnut are deciduous broad-leaved species. Sites 1 and 3 each have three plots, and each plot contains a different monitoring species. Site A1 has one plot containing two different monitoring species, and the remaining sites each have one plot with one monitoring species, giving ten plots in total.

**Table 1:** Description of the sampled trees and monitoring sites.

Site^a^	Species	Age (y)^b^	DBH (cm)^c^	^137^Cs dep.(kBq m^−2^)^d^	Sampling year	
					Bark (whole), wood	Outer bark, inner bark
Site 1 (KU1)	Cedar (*Cryptomeria japonica*)	52	24.2 ± 7.0	747	2011–20	2012–20
	Cypress (*Chamaecyparis obtusa*)	35	20.1 ± 3.2	694	2012–20	2012–20
	Oak (*Quercus serrata*)	35	16.6 ± 4.8	694	2012–20	2016–20
Site 2 (KU2)	Cedar (*Cryptomeria japonica*)	66	35.5 ± 7.9	184	2011–20	2016–20
Site 3 (OT)	Cedar (*Cryptomeria japonica*)	51	27.4 ± 5.6	52	2011–16, 18, 20	2012–16, 18, 20
	Oak (*Quercus serrata*)	52	21.7 ± 5.8	54	2011–17, 19	2015–17, 19
	Pine (*Pinus densiflora*)	52	24.6 ± 6.1	54	2011–16, 19	2012–16, 19
Site 4 (TD)	Cedar (*Cryptomeria japonica*)	47	25.6 ± 5.2	11	2011–16, 20	None
Site 5 (TB)	Cypress (*Chamaecyparis obtusa*)	52	23.0 ± 5.6	33	2011–16, 19	2019
Site A1	Pine (*Pinus densiflora*)	53	26.7 ± 7.1	774	2017–20	2017–20
	Chestnut (*Castanea crenata*)	≈50	25.3 ± 5.5	774	2017–20	2018–20

^a^Site names in parentheses correspond to those in the study of Imamura et al.^31^. ^b^Tree age as of 2020. ^c^Mean ± standard deviation of sampled trees. ^d^Initial deposition density of ^137^Cs following the Fukushima nuclear accident as of July 2, 2011, estimated by Kato et al.^21^. *DBH*, diameter at breast height.

### Sample collection and preparation

Bulk sampling of bark and wood disks was conducted by felling three trees per year at all sites during 2011–2016^8,25^ and at sites 3–5 and A1 during 2017–2020. Partial sampling from six trees per year was conducted at sites 1 and 2 during 2017–2020 (from seven trees at site 2 in 2017) to sustain the monitoring trees. All the samples were obtained from the stems around breast height. During the partial sampling, bark pieces sized approximately 3 cm × 3 cm (axial length × tangential length) were collected from four directions of the tree stem using a chisel, and 12-mm-diameter wood cores were collected from two directions of the tree stem using an automatic increment borer (Smartborer, Seiwa Works, Tsukuba, Japan) equipped with a borer bit (10–101-1046, Haglöf Sweden, Långsele, Sweden). Such partial sampling increases the observational errors in the bark and wood ^137^Cs concentrations in individual trees^26^. To mitigate this error and maintain an accurate mean value of the ^137^Cs concentration, we increased the number of sampled trees from three to six. The sampling was conducted mainly in July–September of each year; the exceptions were site-5 samples in 2011 and 2012, which were collected irregularly during January–February of the following year. The collected bark pieces were separated into outer and inner barks, and the wood disks and cores were split into sapwood and heartwood. The outer and inner bark samples during 2012–2016 were obtained by partial sampling of barks sized approximately 10 cm × 10 cm from 2–3 directions on 2–3 trees per year.

The bulk samples of bark, sapwood, and heartwood were air-dried and then chipped into flakes using a cutting mill with a 6-mm mesh sieve (UPC-140, HORAI, Higashiosaka, Japan). The pieces of the outer and inner bark were chipped into approximately 5 mm × 5 mm pieces using pruning shears, and the cores of the sapwood and heartwood were chipped into semicircles of thickness 1–2 mm. Each sample was packed into a container for radioactivity measurements and its mass was measured after oven-drying at 75 °C for at least 48 h. Multiplying this mass by the conversion factor (0.98 for bark and 0.99 for wood)^8^ yielded the dry mass at 105 °C.

### Radioactivity measurements

The radioactivity of ^137^Cs in the samples was determined by γ-ray spectrometry with a high-purity Ge semiconductor detector (GEM20, GEM40, or GWL-120, ORTEC, Oak Ridge, TN). For measurements, the bulk and partial samples were placed into Marinelli containers (2.0 L or 0.7 L) and cylindrical containers (100 mL or 5 mL), respectively. The peak efficiencies of the Marinelli containers, the 100-mL container, and the 5-mL container were calibrated using standard sources of MX033MR, MX033U8PP (Japan Radioisotope Association, Tokyo, Japan), and EG-ML (Eckert & Ziegler Isotope Products, Valencia, CA), respectively. For the measurement of the 5-mL container, a well-type Ge detector (GWL-120) was used under the empirical assumption that the difference in γ-ray self-absorption between the standard source and the samples is negligible^27^. The measurement was continued until the counting error became less than 5% (higher counting errors were allowed for small or weakly radioactive samples). The activity concentration of ^137^Cs in the bark (whole) collected by partial sampling was calculated as the mass-weighted mean of the concentrations in the outer and inner barks; meanwhile, the concentration in the wood (whole) was calculated as the cross-sectional-area-weighted mean of sapwood and heartwood concentrations. The activity concentrations were decay-corrected to September 1, 2020, to exclude the decrease due to the radioactive decay.

### Trend analyses

The yearly representative values (true states) of ^137^Cs activity concentration in each stem part in each plot were estimated using a DLM, a state-space model in which the noise follows a normal distribution and the relationship between variables is linear. One basic DLM is the local linear trend model defined by the following equations:

1 $$Y_{t} = \mu _{t} + \varepsilon _{t} ,\quad \quad \quad \varepsilon _{t} \sim Normal \left( {0,\sigma _{\varepsilon }^{2} } \right)$$

2 $$\mu_{t} = \mu_{t - 1} + \beta_{t - 1} + \eta_{t} ,\quad \quad \quad \eta_{t} \sim Normal \left( {0,\sigma_{\eta }^{2} } \right)$$

3 $$\beta_{t} = \beta_{t - 1} + \zeta_{t} ,\quad \quad \quad \zeta_{t} \sim Normal \left( {0,\sigma_{\zeta }^{2} } \right)$$

where *Y*_*t*_, *μ*_*t*_, and *β*_*t*_ are the observation values, level (true state), and slope, respectively, and *ε*_*t*_, *η*_*t*_, and *ζ*_*t*_ denote their corresponding noises. The subscript *t* is the time index. The noises *ε*_*t*_, *η*_*t*_, and *ζ*_*t*_ follow normal distributions with a mean of 0 and variances of ${\sigma }_{\varepsilon }^{2}$, ${\sigma }_{\eta }^{2}$, and ${\sigma }_{\zeta }^{2}$, respectively. To detect relatively long-term trends, we employed the smooth local linear trend model^28^ (also called the smooth trend model, integrated random walk model, or second-order trend model), which is obtained by considering that *μ*_*t*_ and *β*_*t*_ are driven by the same noise. The trend changes are assumed to be smoother in this model than in the local linear trend model^28,29^. Combining Eqs. (2) and (3), *μ*_*t*_ in the smooth local linear trend model is finally obtained as

4 $$\mu_{t} = 2\mu_{t - 1} - \mu_{t - 2} + \eta_{t} ,\quad \quad \quad \eta_{t} \sim Normal \left( {0,\sigma_{\eta }^{2} } \right)$$

The parameters *μ*_*t*_, ${\sigma }_{\eta }^{2}$, and ${\sigma }_{\varepsilon }^{2}$ of each stem part in each plot were determined by Bayesian estimation with a Markov chain Monte Carlo (MCMC) method. The Bayesian estimation was performed in R (version 4.1.0)^22^ with the rstan package (version 2.21.2)^30^. Uninformative prior distributions were used for *μ*_*1*_, *μ*_*2*_, ${\sigma }_{\eta }^{2}$, and ${\sigma }_{\varepsilon }^{2}$. The log-transformed values of the ^137^Cs activity concentration (decay-corrected to September 1, 2020) were given as *Y*_*t*_ (the observed values of multiple individuals in each year were passed via the segment function of Stan). MCMC sampling was conducted for four chains of 50,000 iterations (the first 25,000 were discarded as warmup), obtaining 100,000 MCMC samples for each parameter. The MCMC was judged to have converged when the maximum value of *Rhat* was less than 1.05 and the divergent transitions after warmup were fewer than 1,000 (i.e., less than 1% of the MCMC sample size). On the datasets of the outer and inner barks from site-3 oaks and all stem parts from site-A1 pines and chestnuts, the MCMC converged poorly owing to the small number of monitoring years. Thus, the temporal trends in these datasets were not analyzed (the observational data at site A1 are shown in Supplementary Fig. S1 and Table S1).

To detect decadal trends rather than yearly variations, we determined the temporal trends in the true state (*μ*) by setting 2–4 delimiting years and examining whether *μ* varied significantly from one delimiting year to the next. As the delimiting years, we selected the initial and final years of monitoring and the years in which the median *µ* was highest (*µ*-max year) and lowest (*µ*-min year). When the *µ*-max year and/or the *µ*-min year coincided with the initial year and/or final year of monitoring, the number of delimiting years reduced from four to two or three. The trend in *µ* between two delimiting years was determined to be increasing and decreasing when the 95% credible interval of *µ*_2nd delimiting year_ − *µ*_1st delimiting year_ (obtained from the MCMC samples) was higher and lower than zero, respectively. A flat trend (no significant variation) was detected when the 95% credible interval included zero. If the 3rd and 4th delimiting years existed, the trends between the 2nd and 3rd delimiting years and between the 3rd and 4th delimiting years were determined in the same manner.

The ^137^Cs CRs of outer bark/inner bark, heartwood/sapwood, and inner bark/sapwood were also subjected to the above trend analyses. On datasets with less than five years of monitoring, the MCMC did not converge so the trend analysis was not attempted.

## Results

### Temporal trends in bark ^137^Cs concentration

**Figure 2:**
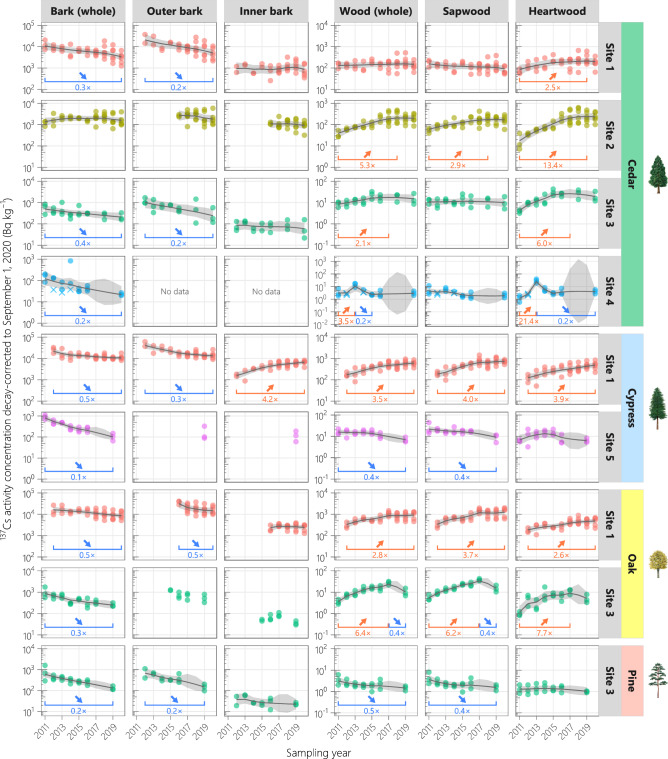
Temporal variations in ^137^Cs activity concentrations (decay-corrected to September 1, 2020) in the bark and wood of the monitoring trees. *Circles* show the observed values, and *crosses* indicate that the ^137^Cs concentration was below the (shown) detection limit. The true states were estimated using a dynamic linear model, and the median values and 95% credible intervals are shown by *solid lines* and *shaded regions*, respectively. *Horizontal lines* indicate significant differences between the estimated true state values in the years at both ends (delimiting years). Data on the bark (whole), wood (whole), sapwood, and heartwood from 2011 to 2016 were provided in previous studies^8,25,31^.

The ^137^Cs activity concentration decay-corrected to September 1, 2020 (hereafter referred to as the ^137^Cs concentration) exponentially decreased in the whole bark samples from all main plots except site-2 cedar (Fig. 2). In the latest monitoring year, the whole bark ^137^Cs concentration in these plots was 0.1–0.5 times that in the initial monitoring year (biological half-life of 2.7–8.7 years). In contrast, the whole bark ^137^Cs concentration in site-2 cedars showed a flat trend during 2011–2020. The decrease rates differed among the sites even for the same species, and no clear species dependency emerged.

**Figure 3:**
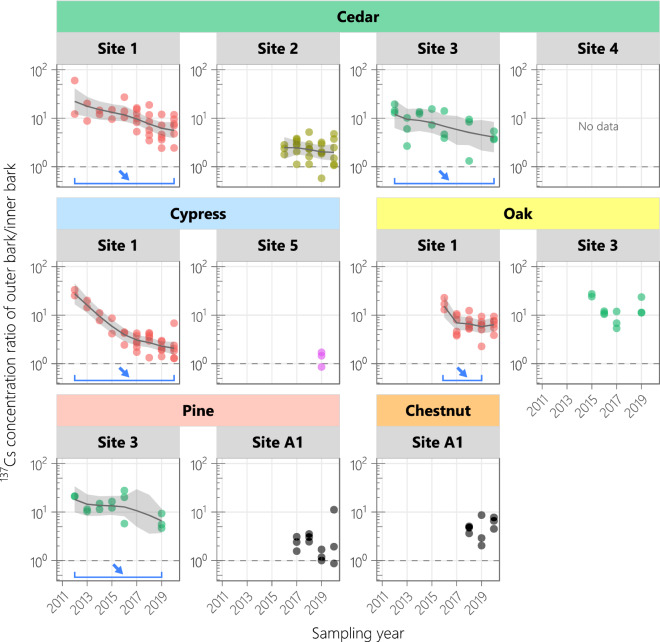
Temporal variations in ^137^Cs concentration ratio of outer bark/inner bark. *Circles* show the observed values. The true states were estimated using a dynamic linear model, and the median values and 95% credible intervals are shown by *solid lines* and *shaded regions*, respectively. *Horizontal lines* indicate significant differences between the estimated true state values in the years at the both ends (delimiting years).

The ^137^Cs concentration in the outer bark showed a decreasing trend (0.2–0.5-fold decrease, biological half-life of 3.2–4.9 years) in all analyzed plots except the site-2 cedar plot (Fig. 2). Although the outer bark monitoring started later than the whole bark monitoring, the ^137^Cs concentration in the outer bark during the monitoring period decreased at comparable or higher rates than that in the whole bark. In contrast, the ^137^Cs concentration in the inner bark did not change significantly in many plots and notably increased (by 4.2-fold) in the site-1 cypress plot. The ^137^Cs CR of outer bark/inner bark exceeded 10 in 2012 and fell to below 10 in most trees by 2020 (Fig. 3). The decrease rate of the CR in the site-1 cypress and oak plots slowed around 2017, and the decreasing trend in the site-1 oak plot shifted to a flat trend. Although the CR in the site-2 cedar plot was not monitored during 2011–2015, it was low (around 2.5) and almost constant since 2016.

### Temporal trends in wood ^137^Cs concentration

The ^137^Cs concentration in the whole wood showed a decreasing trend (0.4–0.5-fold decrease; biological half-life of 6.8–7.2 years) in two of the nine plots, a flat trend in one plot, and an increasing trend (2.1–6.4-fold increase) in six plots (Fig. 2). Among the six plots with increasing ^137^Cs concentrations, the increase leveled off in two plots (site-2 and site-3 cedar) and shifted to a decrease in another two plots (site-4 cedar and site-3 oak). However, when exceptionally high values at the site-4 cedar plot in 2013 were excluded from the analysis, the whole wood ^137^Cs concentration in the site-4 cedar plot remained flat throughout the monitoring period (Supplementary Fig. S2). Whereas cedar and cypress showed different temporal trends at different sites, oak showed the same (increasing) trend at two sites.

**Figure 4:**
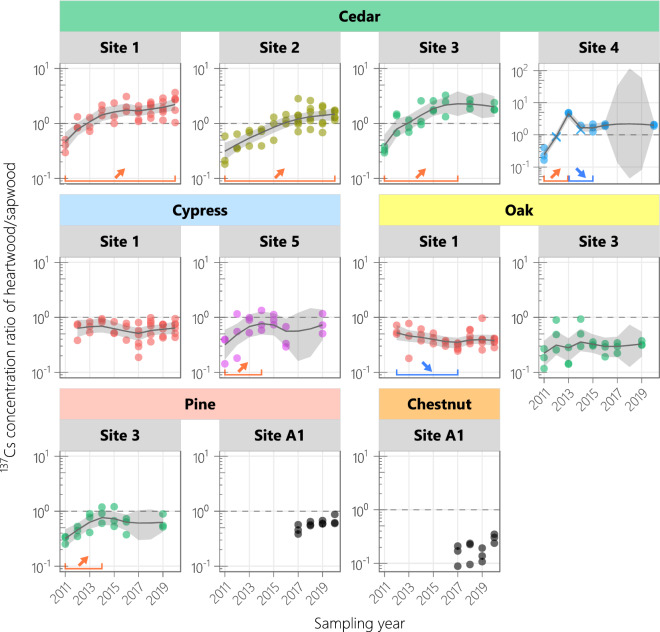
Temporal variations in ^137^Cs concentration ratio of heartwood/sapwood. *Circles* show the observed values, and *crosses* are the calculated values using the detection limits. The true states were estimated using a dynamic linear model, and the median values and 95% credible intervals are shown by *solid lines* and *shaded regions*, respectively. *Horizontal lines* indicate significant differences between the estimated true state values in the years at the both ends (delimiting years). Data from 2011 to 2016 were provided in previous studies^8,25^.

The temporal trends of the ^137^Cs concentrations in sapwood and whole wood corresponded in almost all plots; the exceptions were the site-3 and site-4 cedar plots (Fig. 2). More specifically, the sapwood ^137^Cs concentration decreased by 0.4-fold (biological half-life of 6.3–6.6 years) in two plots, remained flat in three plots, and increased by 2.9–6.2-fold in four plots. In contrast, the ^137^Cs concentration in heartwood remained flat in two plots and increased by 2.5–21.4-fold (or 2.5–13.4-fold excluding the data of the site-4 cedar plot in 2013) in seven plots. A decreasing trend in the heartwood ^137^Cs concentration was observed only in the site-4 cedar plot, where the decrease did not seem like a regular trend (Fig. 2 and Supplementary Fig. S2). The ^137^Cs CR of heartwood/sapwood in cedar increased and eventually exceeded 1.0 at all four sites, whereas those in the other species were typically below 1.0 with no consistent trends (Fig. 4). By 2020, the CR increase had leveled off in four plots but was continuing in the two plots (site-1 and site-2 cedar).

### Temporal trends in inner bark/sapwood ^137^Cs concentration ratio

**Figure 5:**
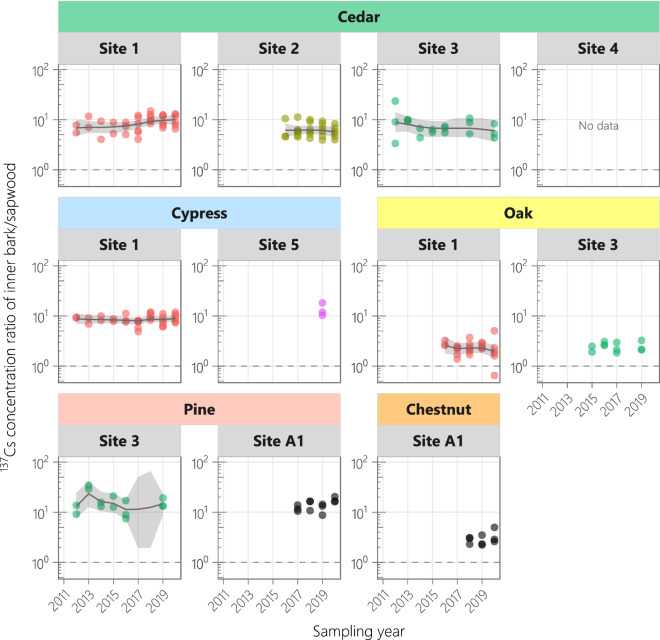
Temporal variations in ^137^Cs concentration ratio of inner bark/sapwood. *Circles* show the observed values. The true states were estimated using a dynamic linear model, and the median values and 95% credible intervals are shown by *solid lines* and *shaded regions*, respectively. Sapwood data from 2011 to 2016 were provided in previous studies^8,25^.

The ^137^Cs CR of inner bark/sapwood, which indicates the ^137^Cs allocation resulting from translocation between the phloem and xylem, showed a flat trend in all analyzed plots over the monitoring period (Fig. 5). The CRs in each species were similar at different sites, with means ± standard deviations of 7.8 ± 1.3 for cedar, 11.0 ± 3.4 for cypress, 15.2 ± 1.5 for pine, and 2.4 ± 0.1 for oak (3.0 for single-site chestnut). The CR was lower in the two deciduous broad-leaved species than in the three evergreen coniferous species.

## Discussion

### Causes of temporal trends in bark ^137^Cs concentration

The ^137^Cs concentration in the whole bark decreased in many plots, clearly because the outer bark ^137^Cs concentration decreased. However, the whole bark ^137^Cs concentration showed a relatively small decrease or even a flat trend in some plots (site-2 cedar and site-1 cypress and oak). In the site-1 cypress plot, where the whole bark ^137^Cs concentration decreased relatively slowly, the inner bark ^137^Cs concentration notably increased. Similarly, although we lack early phase monitoring data in the site-2 cedar and site-1 oak plots, the inner bark ^137^Cs concentration in both plots is considered to have increased prior to monitoring because the sapwood ^137^Cs concentration increased in both plots and the CR of inner bark/sapwood was constant in all other plots. Therefore, the low-rate decrease or flat trend in the whole bark ^137^Cs concentration in some plots was probably caused by an increase in the inner bark ^137^Cs concentration, itself likely caused by high ^137^Cs root uptake (as discussed later).

The ^137^Cs concentration in the outer bark decreased in all four plots monitored since 2012 (site-1 and site-3 cedar, site-1 cypress, and site-3 pine), confirming the ^137^Cs drop/wash off from the bark surface. The constant (exponential) decrease in three of these plots indicates that the ^137^Cs drop/wash off was still continuing in 2020 but with smaller effect on the outer bark ^137^Cs concentration. In contrast, the decrease in the site-1 cypress plot seemed to slow down since around 2017. Furthermore, Kato et al.^32^ reported no decrease in ^137^Cs concentration in the outer bark of Japanese cedar during the 2012–2016 period. Such cases cannot be fitted by a simple decrease of the outer bark ^137^Cs concentration. As a longer-term perspective, in the outer bark of Norway spruces (*Picea abies*) affected by the Chernobyl nuclear accident, the biological half-life of ^137^Cs concentration was extended in areas with higher precipitation, suggesting that high root uptake of ^137^Cs hinders the decreasing trend^33^. The present study showed that 70–80% or more of the ^137^Cs deposited on the bark surface (outer bark) was removed by drop/wash off after 10 years from the accident and that the ^137^Cs CR of outer bark/inner bark became constant in some plots. These facts suggest that the longer-term variations in outer bark ^137^Cs concentration will be more influenced by ^137^Cs root uptake, although it is uncertain whether root uptake caused the slowing down of the decrease rate seen in the site-1 cypress plot. Further studies are needed to understand the ^137^Cs concentration in newly formed outer bark and to determine the ^137^Cs CR of outer bark/inner bark at steady state.

### Causes of temporal trends in wood ^137^Cs concentration

The temporal trends of the ^137^Cs concentration in the whole wood basically corresponded to those in the sapwood. The exceptions were the site-3 and site-4 cedar plots, where the sapwood ^137^Cs concentration did not increase but the whole wood ^137^Cs concentration was raised by the notable increase in the heartwood ^137^Cs concentration. This behavior can be attributed to a species-specific characteristic of Japanese cedar, which facilitates Cs transfer from sapwood to heartwood^8,15,34^. The present study newly found that the increase in the ^137^Cs CR of heartwood/sapwood in the cedar plots became smaller or shifted to a flat trend around 2015–2016, indicating that ^137^Cs transfer between the sapwood and heartwood has reached apparent steady state at many sites 10 years after the accident. Therefore, after 2020, the whole wood ^137^Cs concentration in cedar is unlikely to increase without a concomitant increase in the sapwood ^137^Cs concentration.

The increasing trends in the ^137^Cs concentrations in whole wood and sapwood (site-2 cedar, site-1 cypress, and site-1 and site-3 oak plots) are seemingly caused by the yearly increase in ^137^Cs root uptake; however, the wood ^137^Cs concentration can also increase when the ^137^Cs root uptake is constant or even slightly decreases each year. This behavior can be shown in a simple simulation of the temporal variation in the wood ^137^Cs content (the amount of ^137^Cs in stem wood of a tree). If the ^137^Cs dynamics within a tree have reached steady state and the proportion of ^137^Cs allocated to stem wood become apparently constant, the wood ^137^Cs content in a given year can be considered to be determined by the amount of ^137^Cs root uptake and the amount of ^137^Cs emission via litterfall. The flat ^137^Cs CR trend of inner bark/sapwood during 2012–2020 (see Fig. 5) indicates that the ^137^Cs dynamics, at least those between the inner bark and sapwood, reached apparent steady state within 2011. Here we assume that (1) the annual amount of ^137^Cs root uptake is constant, (2) the proportion of ^137^Cs allocated to stem wood is apparently constant, and as assumed in many forest Cs dynamics models^17,35–37^, (3) a certain proportion of ^137^Cs in the stem wood is lost via litterfall each year. Under these conditions, the simulated amount of ^137^Cs emission balanced the amount of ^137^Cs root uptake after sufficient time, and the wood ^137^Cs content approached an asymptotic value calculated as [*root uptake amount* × *allocation proportion* × (1/*emission proportion* − 1)]. Note that the asymptotic value increases with increasing root uptake amount and decreasing emission proportion and does not depend on the amount of ^137^Cs foliar/bark surface uptake in the early post-accident phase. Nevertheless, the amount of ^137^Cs surface uptake in the early phase critically determines the trend of the wood ^137^Cs content. More specifically, the trend in the early phase will be increasing (decreasing) if the surface uptake is smaller (larger) than the asymptotic value. Finally, the temporal variation of the ^137^Cs concentration in wood is thought to be the sum of the dilution effect of the increasing wood biomass and the above-simulated variation in the wood ^137^Cs content. Therefore, in the early post-accident phase, the wood ^137^Cs concentration will increase when the wood ^137^Cs content increases at a higher rate than the wood biomass. As the wood ^137^Cs content approaches its asymptotic value (i.e., steady state), its increase rate slows and the dilution effect proportionally increases. Then, the wood ^137^Cs concentration shifts from an increasing trend to a decreasing trend. The trends of the ^137^Cs concentrations in whole wood and sapwood in the site-3 oak plot follow this basic temporal trend, which is similarly predicted by many simulation models^9^.

In other plots with the increasing trend (site-2 cedar and site-1 cypress and oak), the increase in the ^137^Cs concentrations in whole wood and sapwood became smaller or shifted to a flat trend around six years after the accident; however, it did not shift to a decreasing trend. This lack of any clear shift to a decreasing trend, which was similarly seen at sites with hydromorphic soils after the Chernobyl nuclear accident^38,39^, cannot be well explained by the above simulation. A core assumption of the simulation that the yearly amount of ^137^Cs root uptake is constant is probably violated in these plots, leading to underestimations of the root uptake amount. Although the inventory of exchangeable ^137^Cs in the organic soil layer has decreased yearly since the accident, that in the mineral soil layer at 0–5 cm depth has remained constant^40^. In addition, the downward migration of ^137^Cs has increased the ^137^Cs inventory in the mineral soil layer below 5-cm depth^41,42^. If the steady state ^137^Cs inventory of the root uptake source can be regarded as sufficient for trees, any increase in the ^137^Cs root uptake is likely explained by expansion of the root distribution and the increase in transpiration (water uptake) with tree growth. When the wood ^137^Cs content increases at a similar rate to the wood biomass, the increasing trend will not obviously shift to a decreasing trend. Therefore, assuming the ^137^Cs allocation and emission proportions in the mature trees do not change considerably with time, the amount of ^137^Cs root uptake is considered to be increasing yearly in these four plots.

In the remaining plots with the decreasing or flat trend (site-1 cedar, site-4 cedar without outliers, site-5 cypress, and site-3 pine), according to the above simulation, the amount of initial ^137^Cs surface uptake was larger than or similar to the asymptotic value, i.e. the amount of ^137^Cs root uptake is relatively small and/or the proportion of ^137^Cs emission via litterfall is relatively high. However, the amount of ^137^Cs root uptake in the plots with the flat trend is possibly increasing because the flat trend has not shifted to a decreasing trend. In these plots, although it is difficult to confirm apparent steady state of the soil–tree ^137^Cs cycling because of the lack of an initial increasing trend, the recent flat trends in the ^137^Cs CRs of heartwood/sapwood and inner bark/sapwood indicate that the ^137^Cs dynamics, at least within the trees, have reached apparent steady state.

Various factors were found to increase the ^137^Cs root uptake after the Chernobyl nuclear accident; for example, high soil water content, high soil organic and low clay content (i.e., low radiocesium interception potential [RIP]), low soil exchangeable K concentration, and high soil exchangeable NH_4_ concentration^12,43^. After the FDNPP accident, the ^137^Cs transfer from soil to Japanese cypress and konara oak was found to be negatively correlated with the soil exchangeable K concentration^44,45^ and the ^137^Cs mobility is reportedly high in soils with low RIP^46^. However, neither the soil exchangeable K and Cs concentrations nor the RIP have explained the different ^137^Cs aggregated transfer factors (defined as [^137^Cs activity concentration in a specified component/^137^Cs activity inventory in the soil]) of Japanese cedars at sites 1–4^46,47^. Because the ^137^Cs dynamics within the forest and trees in many plots reached apparent steady state at 10 years after the FDNPP accident, the ^137^Cs aggregated transfer factor is now considered to be an informative indicator of the ^137^Cs root uptake. Therefore, a comprehensive analysis of the ^137^Cs aggregated transfer factor and the soil properties at more sites than in the present study will be important to understand key factors determining the amount of ^137^Cs root uptake by each tree species at each site.

### Validity and limitation of the trend analyses

Although the application of the smooth local linear trend model failed in plots monitored for less than five years, it was deemed suitable for analyzing the decadal trend because it removes annual noises, which are probably caused by relatively large observational errors (including individual variability)^26^. Moreover, the algorithm that determines the trend and its shift between 2 and 4 delimiting years was apparently reasonable, because the detected trends well matched our intuition. However, when judging a trend, the algorithm simply assesses whether the true state values significantly differ between the delimiting years. Therefore, it cannot detect changes in the increase/decrease rate (i.e., whether an increasing/decreasing trend is approaching a flat trend). For example, the whole bark ^137^Cs concentration in the site-1 cypress plot was determined to decrease throughout the monitoring period. In fact, the decrease rate slowed around 2014 and the decreases were slight between 2014 and 2020 (see Fig. 2). Similarly, the sapwood ^137^Cs concentration in the site-1 cypress and oak plots was determined to increase throughout the monitoring period, but the increase rate has clearly slowed since around 2017. To more sensitively detect the shift from an increasing/decreasing trend to a flat trend, other algorithms are required. Nevertheless, this algorithm is acceptable for the chief aim of the present study; that is, to detect a trend shift from increasing to decreasing.

## Conclusions

In many plots monitored at Fukushima and Ibaraki Prefectures, the ^137^Cs concentrations in the whole and outer bark decreased at almost the same yearly rate for 10 years after the FDNPP accident, indicating that the direct contamination of the outer bark was mostly but not completely removed during this period. Moreover, the ^137^Cs concentration in the whole bark decreased at relatively low rates or was stable in plots where the ^137^Cs root uptake was considered to be high. This fact suggests that indirect contamination through continuous root uptake can reach the same magnitude as direct contamination by the accident.

In all of our analyzed plots, the ^137^Cs CR of inner bark/sapwood has not changed since 2012, indicating that ^137^Cs transfer among the biologically active parts of the tree stem had already reached apparent steady state in 2011. In contrast, the ^137^Cs CR of heartwood/sapwood in six out of nine plots increased after the accident. In four of these plots, the ^137^Cs CR of heartwood/sapwood plateaued after 3–6 years; in the other two plots, the plateau was not reached even after 10 years. Therefore, saturation of ^137^Cs in heartwood (an inactive part of the tree stem) requires several years to more than one decade.

The ^137^Cs concentration in the whole wood showed an increasing trend in six out of nine plots. In four of these plots, the increasing trend shifted to a flat or decreasing trend, indicating that the ^137^Cs dynamics in many forests reached apparent steady state at 10 years after the accident. However, the lack of the clear shift to a decreasing trend indicates that the ^137^Cs root uptake is probably still increasing in some plots. Continuous monitoring surveys and further studies clarifying the complex mechanisms of ^137^Cs root uptake in forests are needed in order to refine the simulation models and improve their prediction accuracy.

## Supplementary Information

Supplementary Information 1.

Supplementary Information 2.

## Data Availability

All data generated or analyzed during this study are included in the present article and its Supplementary Information files.
