# Supplementary material for: Decadal trends in 137Cs concentrations in the bark and wood of trees contaminated by the Fukushima nuclear accident
Source: Sci Rep. 2022 Jul 4;12:11243. doi: 10.1038/s41598-022-14576-1 (PMC9253084; doi:10.1038/s41598-022-14576-1)
Supplement: Supplementary file 1 — Supplementary Information 1. [file 41598_2022_14576_MOESM1_ESM.pdf]

Supplementary Figures

Title

Decadal trends in ^{137}Cs concentrations in the bark and wood of trees contaminated by the Fukushima nuclear accident

Authors

Shinta Ohashi, Katsushi Kuroda, Hisashi Abe, Akira Kagawa, Masabumi Komatsu, Masaki Sugiyama, Youki Suzuki, Takeshi Fujiwara, Tsutomu Takano

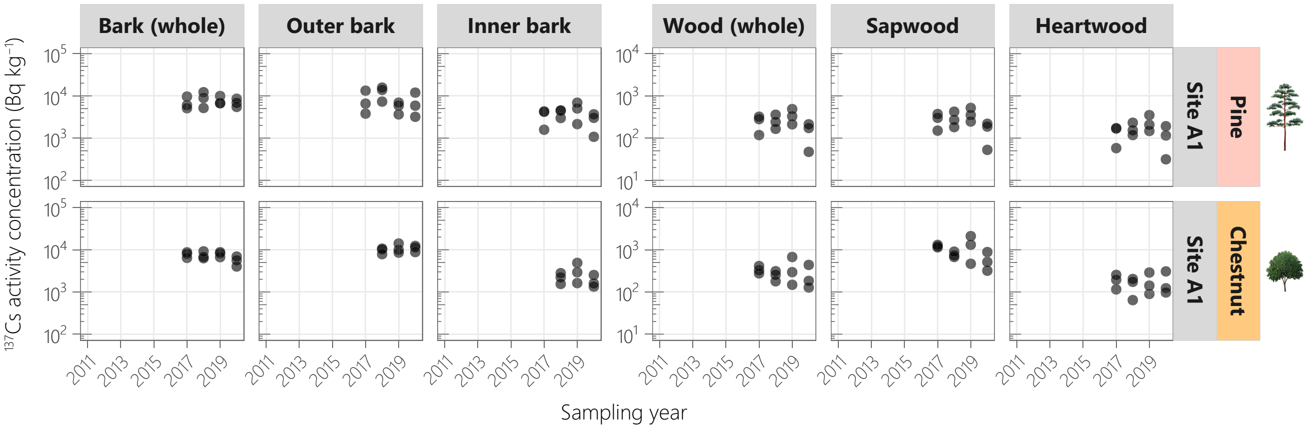

Figure S1. Temporal variations in ^{137}Cs activity concentrations (decay-corrected to September 1, 2020) in the bark and wood of the monitoring trees at site A1. *Circles* show the observed values.

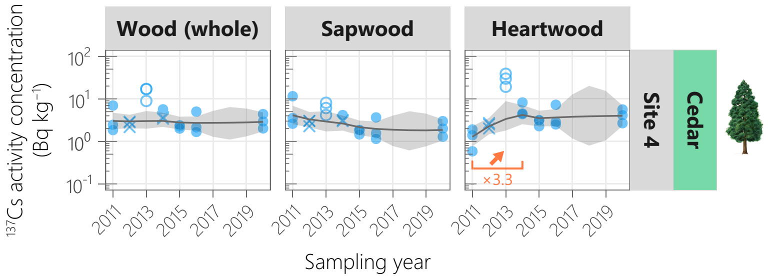

Figure S2. Temporal variations in ^{137}Cs activity concentrations (decay-corrected to September 1, 2020) in the wood parts at site 4, excluding the data of 2013. *Circles* and *open circles* are the observed values and the excluded data, respectively, and *crosses* indicate that the ^{137}Cs concentration was below the (shown) detected limit. The true states were estimated by a dynamic linear model, and the median values and 95% credible intervals are shown by *solid lines* and *shaded regions*, respectively. The *horizontal line* indicates a significant difference between the estimated true values in the years at both ends (delimiting year). Data from 2011 to 2016 were provided in previous studies[8,25,31].
